# Serum levels of FGF21 are reduced and negatively correlated with adiponectin in children with Prader-Willi syndrome

**DOI:** 10.1186/1687-9856-2013-S1-P52

**Published:** 2013-10-03

**Authors:** Su Jin Kim, Young Bae Sohn, Sung-Yoon Cho, Young Ok Choi, Chi Hwa Kim, Dong-Kyu Jin

**Affiliations:** 1 Departmnet of Pediatrics, Kwandong University College of Medicine Myongji Hospital, Goyang, Korea; 2 Department of Medical Genetics, Ajou University Hospital, Suwon, Korea; 3 Department of Pediatrics, Samsung Medical Center, SungKyunKwan University School of Medicine, Seoul, Korea; 4 Clinical Research Center, Samsung Biomedical Research Institute, Seoul, Korea

## Background/Aims

FGF21 (fibroblast growth factor 21) is a novel metabolic regulator that has beneficial effects on glucose homeostasis and insulin sensitivity. In human obesity, serum FGF21 level was increased. The aims of this study were comparing fasting serum levels of FGF21 in Prader-Willi syndrome (PWS) and obese control children and finding correlations these levels with insulin sensitivity and obesity-related parameters.

## Methods

Sixteen children (median age, 10 years; interquartile range, 9.0–13.5 years) with PWS were matched with 16 control subjects (median age, 10.5 years; interquartile range, 9.5- 12.5 years). We measured serum levels of FGF21, adiponectin, insulin sensitivity and obesity-related parameters during oral glucose tolerance test.

## Results

Waist to hip ratio and HOMA-IR were lower in PWS individuals relative to control subjects. Remarkably, serum levels of FGF21 were lower and adiponectin were higher in PWS subjects than in control subjects. FGF21 levels were significantly positively correlated with HOMA-IR and negatively correlated with adiponectin.

## Conclusion

Previously, FGF21 level was reported to increase with obesity. However, compared with obese controls, our results show PWS individuals have lower FGF21 levels. Our data suggest that insulin sensitivity, lower waist to hip ratio, lower FGF21 levels and higher adiponectin levels are the characteristics of PWS children.

